# Medical News

**Published:** 1852-09

**Authors:** 


### MEDICAL NEWS.

*Philadelphia*, Aug. 10th, 1852.

Dear Sir:—The Northern Medical Association have requested me to furnish you with the accompanying resolutions for publication in the Medical Examiner.

J. Henry Smaltz, *Secretary.*

F. Gurney Smith, M. D.

At a Special Meeting of the Northern Medical Association, held Aug. 5th, 1852, the following Preamble and Resolutions were adopted:

*Whereas,* An All-wise Providence has, in his dispensations, seen fit to remove from earth one of the members of our Medical Association;

*Resolved,* That in the death of GEORGE W. PATTERSON, M. D., the members of the Northern Medical Association painfully recognize the loss of a talented and valuable brother, and feel that the community at large has been deprived of a most useful and benevolent citizen.

*Resolved,* That while we bow with proper submission to our great affliction, we will remember and profit by the irreproachable example of upright deportment and sentiment, always evinced by the lamented deceased.

*Resolved,* That while we sincerely mourn this great public and private bereavement, our grief is tempered by the full assurance that our departed friend has entered on a happy immortality.

*Resolved,* That Dr. Joseph R. Bryan be requested to prepare a Biographical Notice of our deceased, lamented member, to be read before the Association.

*Resolved,* That as an evidence of our sympathy with their sorrow, a copy of these our sentiments be transmitted to the family of our late associate.

*Resolved,* That on Saturday next, at 3 o'clock, P. M., the members of this Association will assemble at the usual place of meeting, in order to attend the funeral of our deceased fellow member.

*Resolved,* That a copy of these proceedings be furnished for publication in the Medical Examiner and News, and in one or more of the daily papers.

BENJAMIN S. JANNEY, President.

J. HENRY SMALTZ, Secretary.

DR. JOHN HASTINGS, of San Francisco, California, in a letter to one of the editors, says that he has found a very certain and easy method of introducing Iodine into the system in cases of Phthisis by means of inhalation. A small quantity of dry iodine is placed in a tumbler or cup in the chamber of the patient and allowed to escape by volatilization. He also recommends the introduction of raw or carded cotton into the vagina as a substitute for the pessary. The cotton should be rolled up firmly, so as to fill the vagina, be well anointed, and have a piece of tape secured to it in order to remove it after a few days; after which the vagina should be syringed with cold water and a fresh piece of cotton introduced, and held in situ by a T bandage.

The same gentleman reports two cases of traumatic tetanus successfully treated by the inhalation of Chloroform.

